# Detection of Epizootic Hemorrhagic Disease Virus Serotype 1, Israel

**DOI:** 10.3201/eid2504.180149

**Published:** 2019-04

**Authors:** Natalia Golender, Velizar Y. Bumbarov

**Affiliations:** Kimron Veterinary Institute, Beit Dagan, Israel

**Keywords:** zoonoses, cattle, Orbivirus, Reoviridae, epizootic hemorrhagic disease virus, EHDV, viruses, vector-borne infections, Israel

## Abstract

During September 2016–February 2017, we detected epizootic hemorrhagic disease virus (EHDV) in ruminants in Israel. BLAST and phylogenetic analyses of segment 2 in 6 EHDVs isolated from field samples indicated a close relationship to the EHDV serotype 1 strain in Nigeria. Affected cattle had mostly mild or asymptomatic disease.

Epizootic hemorrhagic disease is an infectious, noncontagious viral disease of ruminants, transmitted by insects of the genus *Culicoides*; it mostly affects white-tailed deer and cattle. Epizootic hemorrhagic disease virus (EHDV) belongs to the genus *Orbivirus* within the family *Reoviridae* and is closely related to bluetongue virus (BTV) and African horse-sickness virus. At least 7 EHDV serotypes are currently recognized worldwide (*1*). (EHDV serotype 1 has been isolated from cattle in the Northern Territory of Australia (*2*), Nigeria, Ecuador, French Guyana, Réunion Island (in the Indian Ocean) (*3*), and the United States, where it was also isolated from white-tailed deer (*4*). In cattle in Israel, EHDV serotype 7 first was recognized in 2006, when it manifested in serious clinical signs (*5*). EHDV serotype 6 caused an outbreak in 2015, when clinical signs were milder than those associated with EHDV serotype 7 (*6*). However, EHDV serotype 6 RNA was found in placenta and brains of aborted cattle fetuses during the latter outbreak (*6*).

In the summer and fall of 2016, several arbovirus infections were registered simultaneously in diseased domestic and wild ruminants in Israel. Included were infections with BTV serotypes 2, 3, 4, 8, and 15; Shuni and Akabane viruses, belonging to the Simbu serogroup of genus *Orthobunyavirus* in the *Peribunyaviridae* family; and EHDV. BTV serotype 8 caused a large outbreak that caused heavy losses of livestock in some sheep and cattle farms.

During September 2016–February 2017, we routinely tested 265 field samples by using specific EHDV real-time reverse transcription PCR (rRT-PCR), as described previously (*6*). The tested field samples included 10 spleen samples from wild and zoo ruminants taken from 4 mountain gazelles, 1 giraffe, 1 Arabian oryx, and 4 Nubian ibexes, as well as 13 spleen samples and 242 whole-blood EDTA samples from diseased cattle. We similarly tested 22 aborted cattle fetuses. We obtained the first EHDV-positive whole-blood sample from a diseased dairy cow on September 12, 2016, and the most recent one on February 26, 2017.

A total of 81 EHDV-positive field samples originated from northern Israel (the Golan Heights, Galilee, the Sharon Plain, and the Jordan Valley) and central Israel (the Coastal Plain). Positive samples included 1 spleen sample from a wild mountain gazelle that was found dead as a result of a head wound near the Sea of Galilee and 2 spleen samples and 78 whole-blood samples from infected cattle. However, the deaths of EHDV-positive cattle probably were not caused by EHDV. In 1 such case, in an adult dairy cow, *Escherichia coli* from all tested internal organs was isolated, and *Babesia* spp. were found during microscopic examination. In another case, in a 4-month-old calf, we identified BTV by using a BTV-specific rRT-PCR test (VetMAX BTV NS3 All Genotypes Kit, LSI; Thermo Fisher Scientific, https://www.thermofisher.com), which led to isolation of BTV serotype 8.

Among the EHDV rRT-PCR–positive samples, 18 were also rRT-PCR–positive for BTV and 1 was positive in pan-Simbu rRT-PCR (*7*), showing Akabane virus infection. All tested aborted cattle fetuses were EHDV-negative by rRT-PCR. We attempted virus isolation on all EHDV- and BTV-positive samples. We isolated 6 EHDV in embryonated chicken eggs, which consequently passed on BHK-21 cells, as previously described (*6*).

We sequenced 4 partial and 2 complete sequences of segment 2 (encoding viral protein 2) by using the standard Sanger method and submitted them to GenBank (accession nos. MG808405–MG808410). BLAST (https://blast.ncbi.nlm.nih.gov/Blast.cgi) and phylogenetic analyses of the segment 2 sequences showed that the EHDV recently isolated in cattle in Israel belongs to serotype 1 and is closely related to the IbAr22619 strain from Nigeria, with which it shares 95.72%–95.76% identity (Figure).

**Figure:**
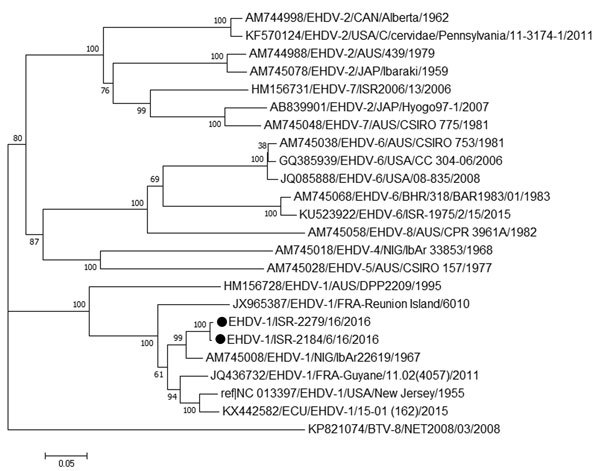
Phylogenetic analysis based on full-length sequences of segment 2 in 2 EHDV serotype 1 isolates from Israel with global EHDVs and BTV-8 from GenBank. We analyzed 24 nucleotide sequences and inferred phylogenetic relationship by using the neighbor-joining method. Numbers below branches indicate bootstrap values. Recent isolates from Israel are marked with black circles. Viruses are identified by GenBank accession number, virus and serotype, 3-letter code of country (and additional information in some cases), isolate identification (in most cases), and year of isolates. Evolutionary analyses were conducted in MEGA7 (*8*). Scale bar indicates number of nucleotide substitutions per site. BTV, bluetongue virus; EHDV, epizootic hemorrhagic disease virus.

Retrospective analysis of clinical signs in EHDV-1–infected cattle enabled us to conclude that in many farms EHDV infection was asymptomatic or subclinical; milk-yield reduction, fever, and recumbency were the only prominent clinical signs observed during the outbreak. However, animals with BTV and EHDV co-infections showed more severe clinical signs, including fever, abortion, lameness, subcutaneous emphysema, and death.

During recent years, several new arboviruses have been detected in Israel that were not identified previously. BTV serotype 3 was first identified in 2016 but probably was present in Israel since at least 2013 (*9*), EHDV serotype 6 was identified in 2015 (*6*), EHDV serotype 1 was found in 2016, and Shuni virus was detected in 2014 (*10*). These findings showed that new introductions of arthropodborne viral infections into the Middle East region had occurred. Molecular epidemiologic data indicate the viruses originated in Africa, as ours and other studies (*5*,*6*) have shown. Molecular diagnostics, vector-control strategies, and epidemiologic studies should be implemented in Israel to mitigate potential risk for future outbreaks.
